# Fusedly Deposited Frequency-Selective Composites Fabricated by a Dual-Nozzle 3D Printing as Microwave Filter

**DOI:** 10.3390/polym16060786

**Published:** 2024-03-12

**Authors:** Jae-Yeon Cho, Young-Chan Oh, Seung-Cheol Shin, Sun-Kon Lee, Hyoung-Seock Seo, Sang-Eui Lee

**Affiliations:** 1 Department of Mechanical Engineering, Inha University, 100 Inha-ro, Michuhol-gu, Incheon 22212, Republic of Korea; 22201204@inha.edu (J.-Y.C.); oyc1104@naver.com (Y.-C.O.); 22201196@inha.edu (S.-C.S.); sun@inha.ac.kr (S.-K.L.); 2 School of Naval Architecture and Ocean Engineering, University of Ulsan, Ulsan 44610, Republic of Korea; 3 Department of Autonomous Vehicle System Engineering, Chungnam National University, 220 Gung-dong, Youseong-gu, Daejeon 34134, Republic of Korea

**Keywords:** dual-nozzle 3D printing, polymer-matrix composites (PMCs), electrical properties, frequency selective surface, transmission loss

## Abstract

We report a fusedly deposited frequency-selective composite (FD-FSCs), fabricated with a dual-nozzle 3D printer using a conductive carbon black (CB) polylactic acid (PLA) composite filament and a pure PLA polymer filament. The square frequency-selective pattern was constructed by the conductive CB/PLA nanocomposite, and the apertures of the pattern were filled with the pure dielectric PLA material, which allows the FD-FSC to maintain one single plane, even under bending, and also affects the resonating frequency due to the characteristic impedance of PLA (*ε*_r_′ ≈ 2.0). The number of the deposition layer and the printing direction were observed to affect electrical conductivity, complex permittivity, and the frequency selectivity of the FD-FSCs. In addition, the FD-FSCs designed for an X-band showed partial transmission around the resonant frequency and was observed to, quite uniformly, transmit microwaves in the decibel level of −2.17~−2.83 dB in the whole X-band, unlike a metallic frequency selective surface with full transmission at the resonance frequency. FD-FSCs embedded radar absorbing structure (RAS) demonstrates an excellent microwave absorption and a wide effective bandwidth. At a thickness of 4.3 mm, the 10 dB bandwidth covered the entire X-band (8.2~12.4 GHz) range of 4.2 GHz. Therefore, the proposed FD-FSCs fabricated by dual-nozzle 3D printing can be an impedance modifier to expand the design space and the application of radar absorbing materials and structures.

## 1. Introduction

Additive manufacturing has garnered attention as an innovative manufacturing technique because it can reduce material waste, save energy, and lower prototyping costs. This method, with manufacturing flexibility, has been developed in a variety of applications including electrochemical energy storage devices (lithium-ion batteries [1], spiral electrodes [2]), biofabrication (tissues and organs [3,4]), aerospace or lightweight structures (gas turbine engine [5], honeycomb [6,7], and lattice [8]), and other research fields. Additive manufacturing depends on the types of materials and additive methods [1,2,3,4,5,6,7,8,9,10]. In fused deposition modeling (FDM) [6,7,8,9,10], one of the representative additive manufacturing methods, a polymeric filament is melted and extruded from a nozzle, and then a designed structure is built up one layer at a time. In general, due to the method of stacking filaments with a circular cross-section one by one, the layers can be clearly counted by observation of microstructures of the cross section or edge of the structure. Physical properties of the 3D-printed structures are observed to be anisotropic in mechanical [9] and thermal [9,10] properties, depending on the printing and stacking direction.

When detecting an aircraft, radars sense electromagnetic (EM) signals backscattered from the targets. Radar cross-section (RCS), a measure of the size of the reflected radar signal [11], should be minimized in stealth technology. Radar cross-section reduction (RCSR) technology has become an important factor in protecting aircraft from radar detection. The radar cross-section reduction (RCSR) technology includes the shaping of targeting objects, reflecting the radar signal out of the radar range and also the material design covering the object surface absorbing radar signals [12,13,14,15,16].

The design of the material includes radar absorbing materials (RAMs) [12,13,14] with dielectric, electrically conductive, or magnetic particles in polymeric binders, radar absorbing structures (RASs) with fiber-reinforced composites with carbon fibers or glass fibers [15,16], and frequency selective surfaces (FSSs) [17,18] consisting of a periodic pattern made of a conductive material represented by metals using chemical etching [17,18,19], particulate composites by coating, (3D) printing, and impregnating [20,21,22,23,24,25], and textile composites with hybrid continuous glass or carbon fibers [25,26]. The additive manufacturing can be utilized to realize the material approaches for RCS reduction [22,23,24,27,28,29].

The FSS has a periodic surface structure that transmits, reflects, or absorbs EM waves in the resonant frequency band [17,18]. The inductive FSS, with a periodic aperture, passes microwaves at around the resonant frequency, while the capacitive FSS with a periodic metallic patch reflects EM waves for the corresponding resonant frequency. Since the transmission/absorption frequency band changes according to the pattern geometry, various studies have been made for designing and tailoring the transmission and the absorption of EM waves [17,18].

With the advantage of additive manufacturing for the quick prototyping of complex shape structures, FSSs have been designed and their characteristics were investigated. Composite filaments of copper particles dispersed in polylactic acid were used to print a FSS with circular apertures of 2.13 mm in thickness, 20.6 mm in diameter, and 28 mm in between, on a 4 mm Rohacell foam substrate, and the FSS showed a stop band characteristic as inductive FSS in the X-band [22]. A mixture of a vinyl polymer and a carbohydrate was 3D-printed to make the FSS of silver-coated dipole elements folded in the volume of 13 × 13 × 21 mm^3^, and the FSS possessed resonant frequencies (*f*_res_) at around 2 GHz and 3.2 GHz for different orthogonal planes, respectively, due to its three dimensionality [23]. Another composite filament composed of flaky carbonyl iron particles (FCIPs) and polyether-ether-ketone (PEEK) was used to fabricate a complex-gradient FSS with the thickness of 10 mm and −10 dB absorbing bandwidth in the broadband of 5.1 to 40 GHz [24].

Aside from the FDM-based FSS, there were studies of FSSs with a combination of types of additive manufacturing–materials, such as inkjet printing–silver nanoparticle ink on polyester cotton fabric square aperture of 28 × 28 mm^2^ as unit cell and 0.5 mm in thickness for *f*_res_~3 GHz [27], stereolithography apparatus (SLA)–Al_2_O_3_/SiC whisker (SiCw) by chemical vapor infiltration (CVI) honeycomb pattern with a diameter of 0.5 mm and a wall height of 3.5 mm for −10 dB absorbing bandwidth in the whole X-band (8.2~12.4 GHz) [28], and SLA–a resin (with a permittivity of 2.4) gradient-refractive-index radar absorbing structure (GRIN-RAS) of the outer and inner radii of 109 mm and 58 mm, respectively, for −10 dB absorbing bandwidth in the Ku-band by multi-layer structuring with a ferromagnetic rubber sheet [29]. 

**Figure 1:**
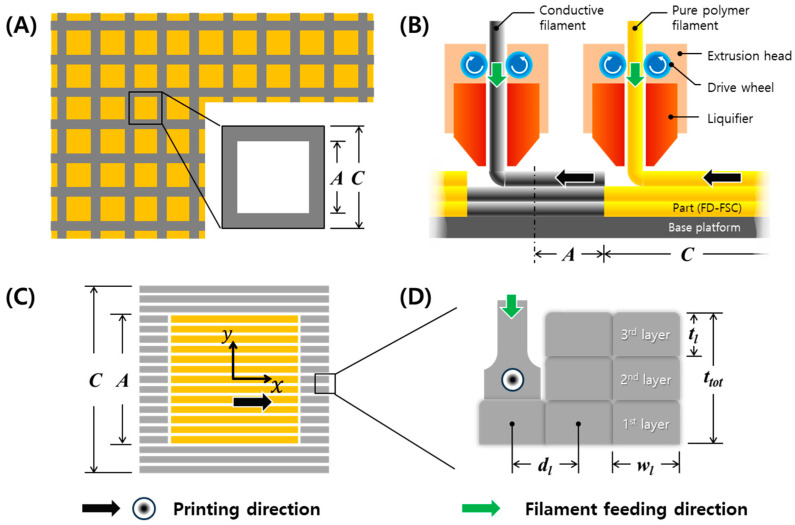
Schematics of (**A**) square frequency selective surface (FSS), (**B**) dual nozzle printing, (**C**) PLA-aperture fusedly deposited frequency selective composite (FD-FSC), and (**D**) multiple layer stacking by 3D printing, with printing and filament feeding directions.

In this paper, a fusedly deposited frequency-selective composite (FD-FSC) with a square pattern shown in Figure 1A was designed for X-band microwave absorption, and it was fabricated with the fused deposition modeling using carbon black (CB) polylactic acid (PLA) composite filament. The characteristics of FD-FSC were investigated in terms of properties of the deposited composites and the number of printed layers, and the effect of filling in the aperture was also evaluated. Additionally, RAS with a FD-FSC embedded in pure PLA layers were devised and its reflection loss was analyzed.

## 2. Materials and Methods

### 2.1. Materials

The conductive filament (CDP1xxxx, Proto-pasta, US) suited for 3D printing was used in this study, containing the conductive carbon particles and PLA resin. The weight content of the carbon particles is 21.4 wt%. The filament was made in a way that CB particles and PLA resin were first put into an injection machine, and then the materials were melted and mixed through an auger screw extrusion. The composite (CB/PLA) filament was melted at 195–225 °C and its relative density was 1.24 g/cc. The printing temperature was setup at 250 °C. For the aperture region generated by the conductive filament printing, a pure PLA filament (PLA+ filament, Shenzhen Esun Industrial Co., Ltd., Shenzhen, China) was used.

### 2.2. Preparation of FD-FSC

A square aperture FD-FSC was fabricated by FDM and its size of aperture (*A*) and the unit cell (*C*), in Figure 1A was calculated by Equation (S3). FDM is a type of material extrusion additive manufacturing, according to the ISO/ASTM standards. In Table S1, *A* = 16.8 mm, *C* = 24.0 mm for *f_res_* = 11.7 GHz. The air-aperture FD-FSC and the PLA-aperture FD-FSC were fabricated by dual-nozzle printing, as shown in Figure 1B,C.

The processing parameters of the 3D printing are summarized in Table 1 and shown in Figure 1B, including the infill setting, temperature, condition of retraction, and printing. The infill settings affect the thickness and width of a line or layer. The line width (*w_l_*) and infill line distance (*d_l_*) were set as 0.8 mm and 0.6 mm in order to tightly contact the infill lines. The line or layer thickness (*t_l_*) was measured 0.2 mm on average.

Top and side views of the square-aperture FD-FSC were shown in Figure 1C,D. In Figure 1C, the pattern fabrication was carried out in a one-printing direction. The directions parallel and perpendicular to the printing direction were set up as *x*-direction and *y*-direction, respectively. As shown in Figure 1D, FD-FSCs were fabricated with the number of layers, *n* = 1, 3, 5, 7, and 9, respectively. The five types of the FD-FSCs were prepared by the dimension of 150 mm × 150 mm for measurement of microwave property with the constant printing speed of 30 mm/s.

### 2.3. Measurement

In order to investigate effects of the printing direction to material properties [30], microstructures of the cross section in the x- and y-directions of the FD-FSCs were observed by using a scanning electron microscopy (S-4300SE, HITACHI, Tokyo, Japan). The printed FSCs were fractured after submerging in liquid nitrogen.

The electrical conductivity of the five types of FD-FSCs, with regard to the number of layers or the total thicknesses, was measured in the *x*- and *y*-directions using a digital multimeter 34465A (Keysight, Santa Rosa, CA, USA), respectively. A silver paste, ACH35001LV (Protavic Korea, Daejeon, South Korea) was used as an electrode, and it was coated on the surface of the FD-FSCs and cured in an oven at 90 °C for 30 min and then 175 °C for 1 h.

To measure permittivity of FSC, the specimens were fabricated in the dimension of 150 mm × 150 mm with the different number of layers, for an example, 0.6 mm in thickness for the three-layer printing. The free space measurement system was employed to measure the transmission loss of the transverse electromagnetic (TEM) waves, and it has a pair of spot-focusing horn-lens antennas, a network analyzer (HP 8510C), and a data acquisition computer system. The specimens were placed at the center of the space between the two horn-lens antennas. For the minimization of multiple reflections, time-domain gating of the network analyzer and TRL (through-reflect-line) calibration were used [15,25].

## 3. Results and Discussion

### 3.1. Microstructure of Conductive FD-FSC

**Figure 2:**
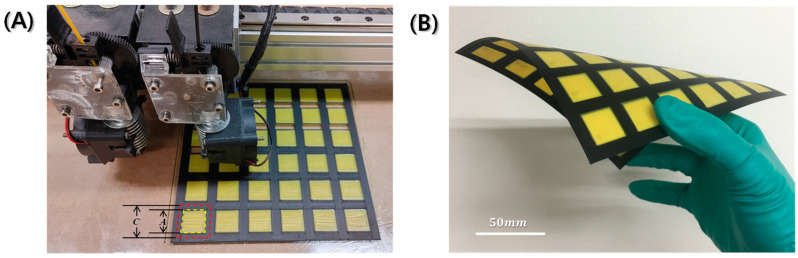
Photographs of (**A**) PLA-aperture FD-FSC under dual nozzle 3D printing process, and (**B**) fabricated FD-FSC under bending.

**Figure 3:**
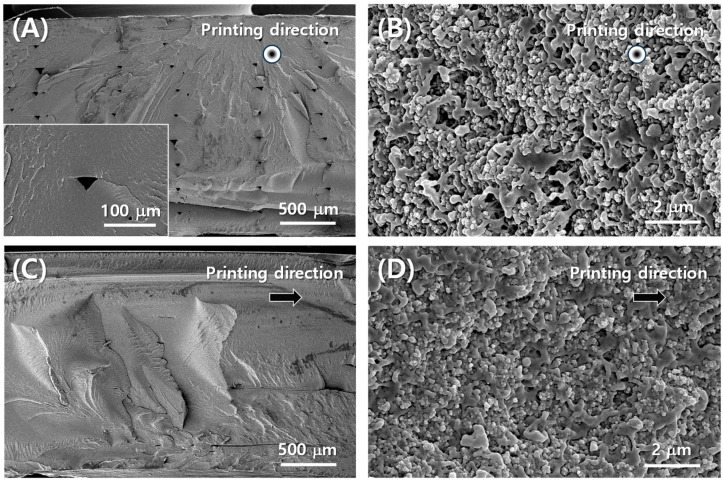
SEM images of cross section of conductive filament part of FD-FSC (**A**,**B**) parallel to printing direction, (**C**,**D**) vertical to printing direction.

Figure 2A shows the PLA-aperture FD-FSC under fabrication with the dual nozzle 3D printing equipment. In Figure 2A, there is a yellow filament in the left nozzle. This is the pure polymer filament entering the extrusion head, while the other nozzle contains the black conductive filament. Figure 2B shows that once fabricated, it can be flexible with a single curvature by filling in the aperture area, while that air aperture one can’t be. Figure 3 displays microstructures in the directions parallel (in Figure 3A,B) and perpendicular (in Figure 3C,D) to the printing direction. The fractured surface was prepared by Figure 1D and was drawn as schematic of Figure 3A. The upper surface of a layer has round edges, while the lower surface is flat, and thus the pore shape is like a tent-like triangle in Figure 3A in the printing direction, whereas such a pore shape was not observed in the perpendicular direction, as shown in Figure 3B. The pore size could be minimized by adjusting the infill line width (*w_l_*) and distance (*d_l_*), in comparison with a 3D-printed heater case [30]. Figure 3B,D show CBs are well distributed in the PLA resin. The interfacial adhesion of CB particles and the resin is observed to be needing enhancement, and a proper surface treatment of the carbon particles can be adopted [31].

### 3.2. Anisotropic Characteristics of Electrical Properties

**Figure 4:**
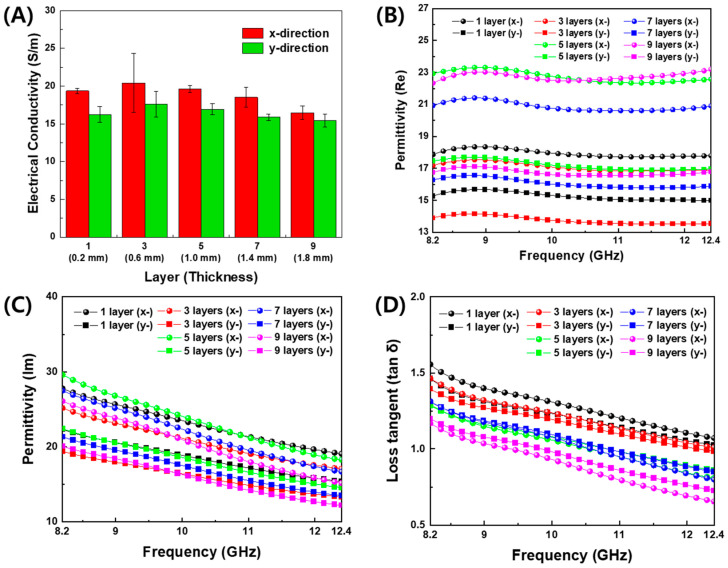
Material properties of 3D-printed plates with conductive CB/PLA filament. (**A**) Electrical conductivity, (**B**) real part, (**C**) imaginary part, and (**D**) loss tangent of permittivity, with regard to frequency, thickness, and printing direction.

The measured electrical conductivity and permittivity of PLA and conductive CB/PLA composites is shown in Figure 4. Both electrical conductivity and permittivity of 3D-printed FSS were found to be anisotropic. In Figure 4A, the electrical conductivity in the *x*-direction (*σ_x_*) is higher than in the *y*-direction (*σ_y_*) on the same thickness. *σ_x_* and *σ_y_* are in the ranges of 16–21 S/m and 15–18 S/m, respectively. Electrical anisotropy may be attributed to the alignment and networking of the conductive CB particle along the printing flow direction. For the *y*-direction perpendicular to the printing direction, the CB particles can be connected in inter-filament paths [30,32]. Different types of carbon particles, carbon nanotubes, carbon fibers [9], and graphene [33], are also observed to be aligned in the 3D-printing direction, which induces the anisotropic nature due to the different degrees of interdiffusion and re-entanglement of the polymer chains and the alignment of conductive particles along the in-plane and out-of-plane directions [9].

The real and imaginary parts of the permittivity of the conductive filament printing plates with regard to frequency are displayed in Figure 4B–D. As shown in Figure S1, the measured values of the real permittivity of PLA were 1.94~1.99, and the dielectric loss tangent was 0.005~0.05 in the X-band. In Figure 4B, the real permittivity was observed to be constant regardless of frequency. The imaginary permittivity in Figure 4C and the tangent loss in Figure 4D gradually decreased with increasing frequency. This tendency is consistent with the result of the electrical conductivity, because the electrical conductivity is proportional to the imaginary permittivity [15].

**Figure 5:**
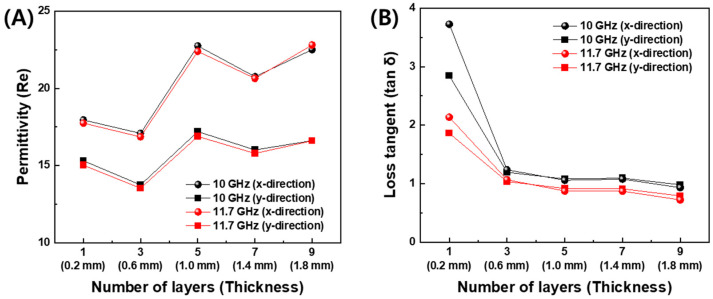
Permittivity of 3D-printed plates with conductive CB/PLA filament. (**A**) Real part and (**B**) dielectric loss tangent at 10 GHz and 11.7 GHz.

The dependence of the permittivity on the number of the deposition layer is displayed at 10 GHz and 11.7 GHz in Figure 5. The latter corresponds to the resonant frequency of the designed FD-FSCs. As the number of the deposition layer increased, the real permittivity increased in Figure 5A, while the imaginary part decreased at the one single layer deposition. Then, in the almost constant level after three-layer printing, as shown in Figure 5B. In addition, the printing direction or *x*-direction showed higher values of permittivity than those in the out-of-printing direction or *y*-direction. This trend can be attributed to the degree of interdiffusion, alignment, and entanglement of the polymer chains, as well as the degree of formation of conductive path regarding printing direction [9,15,32,33].

### 3.3. Transmission Loss of FD-FSC

**Figure 6:**
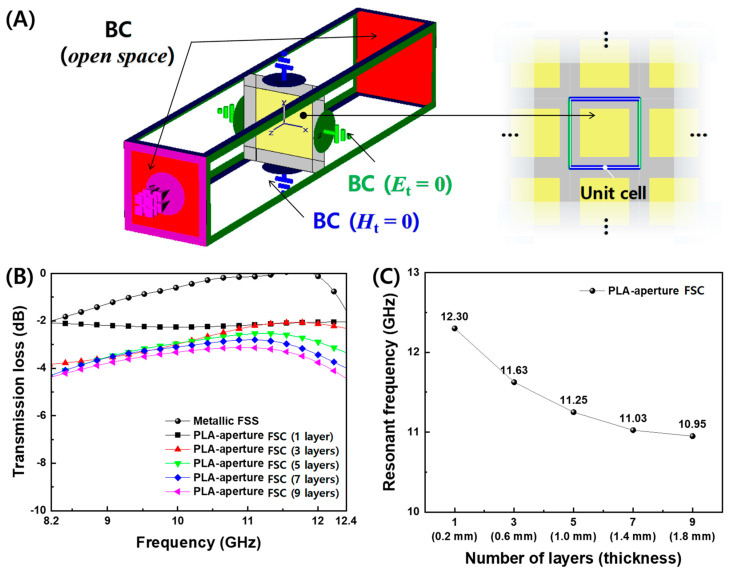
(**A**) Schematic of boundary conditions of microwave simulation using the unit cell of FD-FSCs, and the simulated results of (**B**) the transmission losses and (**C**) the resonant frequencies for the designed PLA-aperture FD-FSCs with regard to the number of layers.

Based on the measured electrical conductivity and permittivity of PLA and CB/PLA composites in Figure 4 and Figure 5, the transmission loss of FD-FSCs, as designed in Table S1, with the sizes of aperture (*A* = 16.8 mm) and unit cell (*C* = 24.0 mm) for *f_res_* = 11.7 GHz was simulated by a commercial software, CST Microwave Studio (CST-MWS, CST Studio Suite 2021). The FD-FSCs with the number of deposition layers was modeled with a unit cell by placing a periodic boundary condition on four side surfaces of the simulation space, zero tangential magnetic fields on two facing surfaces, and zero tangential electric fields on the other two, as shown in Figure 6A. As shown in Figure 6B, the metallic FSS shows that the transmission loss is 0 dB, that is, full transmission, at the resonant frequency of 11.7 GHz. Unlike the metallic FSS, the PLA-aperture FSCs showed a partial transmission of microwaves around the resonant frequency. In addition, the resonant frequencies decreased as the number of the deposition layers increased, as shown in Figure 6C. The resonant frequency is defined as frequency corresponding to the minimum transmission. It can be attributed to the lower electrical conductivity of CB/PLA composites than that of a metal (~10^7^ S/m) and also to the decrease in the electrical conductivity while increasing the number of the layers. The carbon fiber-based frequency selective fabric composite with the aperture filled with glass fiber/epoxy composite showed the similar partial transmission near the resonant frequency due to its electrical conductivity of 3.5 × 10^4^ S/m [26]. The unique EM characteristic represented by the partial transmission around the resonant frequency can make the FD-FSCs an novel impedance modifier, and they can be incorporated with or embedded in RAM [12,13,14] or RAS [15,16,25].

**Figure 7:**
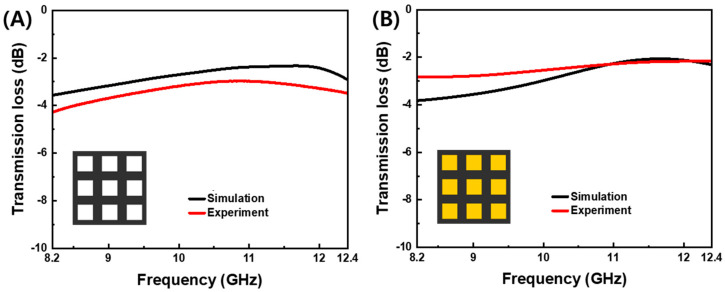
Simulated and measured transmission losses vs. frequency of (**A**) air-aperture FD-FSC and (**B**) PLA-aperture FD-FSC (where the yellow color is the PLA filling) for the three-layer deposition (0.6 mm in thickness) for the design parameters of the aperture size (*A* = 16.8 mm) and unit cell (*C* = 24.0 mm).

The PLA-aperture FD-FSC and the air-aperture FD-FSC were compared for the three-layered configuration. As shown in Figure 7, the measured transmission losses were in good agreement with the simulated ones in both cases. The PLA-aperture FD-FSC does not allow the microwaves near *f*_res_ to be transmitted over the the air-aperture FD-FSC, but conversely the transmission is in the lower frequency, at around 8.2 GHz. The dielectric loading effect in the aperture area can be figured out in the viewpoint of characteristic impedance of air (*ε*_r_′ = 1) and PLA (*ε*_r_′ ≈ 2.0) and impedance mismatching [17,25].

### 3.4. Electromagnetic Wave Absorption of FD-FSCs

**Figure 8:**
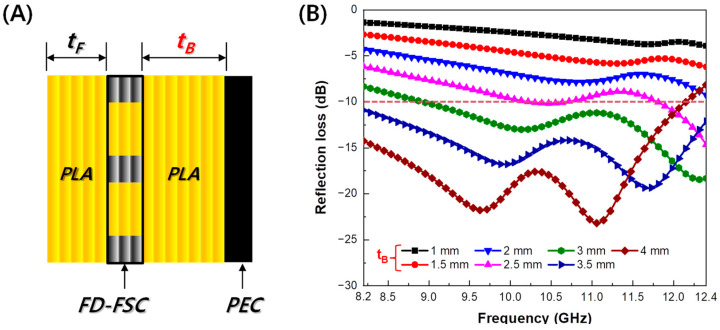
(**A**) Schematic of FD-FSC-embedded RAS and (**B**) the simulated results of reflection loss.

The FD-FSC embedded Radar Absorbing Structure (RAS) is a multi-layered structure consisting of Front-PLA, FD-FSC, and Back-PLA. The thickness of Front-PLA and FD-FSC is 0.2 mm (*t_F_*) and 0.6 mm (3 layers), respectively. The thickness of Back-PLA (*t_B_*) was optimized (shown in Figure 8A). Figure 8B illustrates the reflection loss curves for FD-FSC embedded RAS with varying thicknesses of Back-PLA. As depicted in Figure 8B, there is a noticeable improvement in the microwave absorption performance of the Radar Absorbing Structure (RAS), with an increase in the thickness of Back-PLA from 1.0 mm to 3.5 mm. Specifically, when the Back-PLA thickness is 3.5 mm, the reflection loss values below −10 dB are observed in the X-band (8.2~12.4 GHz). This indicates a 10 dB bandwidth of 4.2 GHz. However, with a Back-PLA thickness of 4.0 mm, certain regions exhibit reflection loss values below −20 dB. Nevertheless, in the vicinity of 12.4 GHz, a loss of more than −10 dB is observed, and there is a slight decrease in the 10 dB bandwidth.

**Figure 9:**
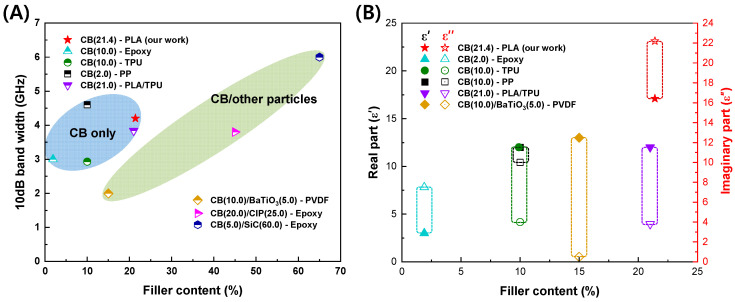
(**A**) 10 dB bandwidth and (**B**) complex permittivity according to filler content of the carbon black-based composites.

The microwave absorption properties of CB-based composites are compared and presented in Table 2. The FD-FSC-embedded Radar Absorbing Structure (RAS) in this study, despite having a somewhat thicker thickness, exhibits strong absorption performance compared to other CB-based structures with similar content. Further details regarding the property comparison based on filler content are illustrated in Figure 9A,B. Additionally, in Figure 9, the particle weight percentages are indicated within parentheses.

Figure 9A represents the 10 dB Bandwidth (BW) according to filler content. Only when CB is used as the filler, the FD-FSC-embedded Radar Absorbing Structure (RAS) demonstrates a stronger absorption performance compared to other structures. The variations in performance and properties at similar content levels can be attributed to the characteristics of CB (composition, content, shape, degree of agglomeration, and electromagnetic properties) [34], as well as the characteristics of the matrix (chemical structure, content, electromagnetic properties, viscosity, surface energy) and the processing factors affecting dispersion and fabrication of films or composites, including filler alignment [35,36]. Figure 9B illustrates the complex permittivity according to filler content. In most studies, regardless of the filler content, the real part tends to exhibit similar values. However, it is notable that the imaginary part of FD-FSC is significantly higher compared to other studies. According to the free electric theory [37], an imaginary part could be obtained to be *ε″* ≈ *σ*/*2πfε*_0_, where *σ* is electrical conductivity, *f* is frequency and *ε*_0_ is the dielectric constant in vacuum. As observed in Figure 3, the even dispersion of CB in FD-FSCs seems to form a well-established conductive network, contributing to its elevated *ε″*. Generally, the real and imaginary parts of permittivity of CB composites are observed to increase as the content of CB increases [16,38,39,40], which is consistent with the material behaviors shown in MWNT-loaded GF/EP plain weave composite [15], CB/EP-based glass fabric composite [16], CB/TPU-based composite sheet [38], CB/PP(Polypropylene) composites [39], and CB/PLA/TPU (Thermoplastic polyurethane) composites [40].

**Table 2:** Comparison of microwave absorption properties of carbon black-based composites.

Filler			Polymer Matrix	Thickness (mm)	Permittivity (at 10 GHz)	10 dB BW (GHz)	Ref.
	Type	Wt%					
CB only	CB	21.4	PLA	4.3	17.1–j22.2	4.2 (8.2–12.4)	Our work
	CB	2.0	Epoxy	2.7	3.0–j7.5	3 (8.5~11.5)	[16]
	CB	10.0	TPU	1.8	12.0–j4.0	2.9 (11.1~14.0)	[38]
	CB	10.0	PP	2.8	12.0–j10.0	4.6 (8.8~13.4)	[39]
	CB	21.0	PLA/TPU	2.57	12.0–j3.8	3.8 (8~11.8)	[40]
Binary Particles	CB/BaTiO_3_	10.5/5.0	PVDF	2.5	13.0–j0.5	2.0 (8.3~10.3)	[41]
	CB/CIP	20.0/25.0	Epoxy	1.5	-	3.8 (14.2~18)	[42]
	CB/SiC	5.0/60.0	Epoxy	2.0	-	6.0 (6.4~12.4)	[43]

## 4. Conclusions

The fusedly deposited frequency selective composites (FD-FSCs) were fabricated by the dual-nozzle 3D printing. Inductive types of FD-FSCs with a square aperture were investigated using a conductive CB/PLA filament and a pure PLA filament. The dual-nozzle 3D printing made it possible for the FD-FSCs to be flexible with a single curvature. FD-FSCs were found to have a frequency selectivity depending on the printing direction and the number of the deposition layers, but they can also have a partial transmission around the resonant frequency. A PLA-aperture FD-FSC with a three-layer deposition was demonstrated to evaluate the transmission modification, and it was observed to quite uniformly transmit microwaves in the decibel level of −2.17~−2.83 dB in the whole X-band. FD-FSCs embedded with RAS exhibited excellent microwave absorption and a wide effective bandwidth. At a thickness of 4.3 mm, the 10 dB bandwidth covered the entire X-band range of 4.2 GHz (8.2~12.4 GHz), showcasing an outstanding performance. It may be concluded that the FD-FSCs, covering inductive types with an aperture, as well as capacitive types with a conductive patch, can function as microwave filter or impedance modifier and can be applied to RAMs or RASs to broaden microwave absorption.

## Figures and Tables

**Table 1:** Printing parameters of the single nozzle 3D printer.

	Infill Settings		Retraction			Printing		
Specimen	Line Thickness *t_l_* (mm)	Line Width *w_l_* (mm)	Infill Line Distance *d_l_* (mm)	Distance (mm)	Speed (mm/s)	Infill Density (%)	Speed (mm/s)	Number of Print Layers
1 layer	0.2	0.8	0.6	6.7	25	100	30	1
3 layers	0.2	0.8	0.6	6.7	25	100	30	3
5 layers	0.2	0.8	0.6	6.7	25	100	30	5
7 layers	0.2	0.8	0.6	6.7	25	100	30	7
9 layers	0.2	0.8	0.6	6.7	25	100	30	9

## Data Availability

Data are contained within the article and Supplementary Materials.

## Supplementary Materials

The following supporting information can be downloaded at: https://www.mdpi.com/article/10.3390/polym16060786/s1, Equations (S1)–(S3): Equations for resonant frequency of a metallic FSS with square pattern; Table S1: Design parameters and resonant frequency of the metallic FSS; Figure S1: Measured permittivity of PLA. (A) Real and imaginary parts of permittivity, and (B) dielectric loss tangent.

## References

[1] Reyes C., Somogyi R., Niu S., Cruz M.A., Yang F., Catenacci M.J., Rhodes C.P., Wiley B.J. (2018). Three-dimensional printing of a complete lithium ion battery with fused filament fabrication. ACS Appl. Energy Mater..

[2] Ambrosi A., Moo J.G.S., Pumera M. (2015). Helical 3D-printed metal electrodes as custom-shaped 3D platform for electrochemical devices. Adv. Funct. Mater..

[3] Zadpoor A.A., Malda J. (2016). Additive manufacturing of biomaterials, tissues, and organs. Ann. Biomed. Eng..

[4] Murphy S.V., Atala A. (2014). 3D bioprinting of tissues and organs. Nat. Biotechnol..

[5] Ngo T.D., Kashani A., Imbalzano G., Nguyen K.T.Q., Hui D. (2018). Additive manufacturing (3D printing): A review of materials, methods, applications and challenges. Compos. Part B Eng..

[6] Jiang W., Yan L., Ma H., Fan Y., Wang J., Feng M., Qu S. (2018). Electromagnetic wave absorption and compressive behavior of a three-dimensional metamaterial absorber based on 3D printed honeycomb. Sci. Rep..

[7] Ainin F.N., Azaman M.D., Majid M.S.A., Ridzuan M.J.M. (2023). Investigating the low-velocity impact behaviour of sandwich composite structures with 3D-printed hexagonal honeycomb core—A review. Funct. Compos. Struct..

[8] Praveen Kumar A., Ma Q. (2023). Evaluation of energy absorption enhancement of additively manufactured polymer composite lattice structures. Funct. Compos. Struct..

[9] Chen J., Liu X., Tian Y., Zhu W., Yan C., Shi Y., Kong L.B., Qi H.J., Zhou K. (2021). 3D-Printed Anisotropic Polymer Materials for Functional Applications. Adv. Mater..

[10] Stepashkin A.A., Chukov D.I., Senatov F.S., Salimon A.I., Korsunsky A.M., Kaloshkin S.D. (2018). 3D-printed PEEK-carbon fiber (CF) composites: Structure and thermal properties. Compos. Sci. Technol..

[11] Knott E.F. (2012). Radar Cross Section Measurements.

[12] Wu N., Hu Q., Wei R., Mai X., Naik N., Pan D., Guo Z., Shi Z. (2021). Review on the electromagnetic interference shielding properties of carbon based materials and their novel composites: Recent progress, challenges and prospects. Carbon.

[13] Lim G.-H., Woo S., Lee H., Moon K.-S., Sohn H., Lee S.-E., Lim B. (2017). Mechanically robust magnetic carbon nanotube papers prepared with CoFe_2_O_4_ nanoparticles for electromagnetic interference shielding and magnetomechanical actuation. ACS Appl. Mater. Interfaces.

[14] Lee J., Lee K., Kim T., Lee S.B. (2019). Enhanced microwave absorption properties of graphene/FeCoNi composite materials by tuning electromagnetic parameters. Funct. Compos. Struct..

[15] Lee S.-E., Kang J.-H., Kim C.-G. (2006). Fabrication and design of multi-layered radar absorbing structures of MWNT-filled glass/epoxy plain-weave composites. Compos. Struct..

[16] Kim J.B., Lee S.-K., Kim C.-G. (2008). Comparison study on the effect of carbon nano materials for single-layer microwave absorbers in X-band. Compos. Sci. Technol..

[17] Munk B.A. (1995). Frequency Selective Surfaces and Grid Arrays.

[18] Munk B.A. (2000). Frequency Selective Surfaces: Theory and Design.

[19] Shung-Wu Lee G. (1982). Zarrillo and Chak-Lam Law. Simple formulas for transmission through periodic metal grids or plates. IEEE Trans. Antennas Propag..

[20] Panwar R., Puthucheri S., Agarwala V., Singh D. (2015). Fractal frequency-selective surface embedded thin broadband microwave absorber coatings using heterogeneous composites. IEEE Trans. Microw. Theory Tech..

[21] Xu C., Duan G., Xu W., Wang X., Huang Y., Zhang X., Zhu H., Wang B.-X. (2023). Thermally tunable vanadium-dioxide-based broadband metamaterial absorber with switchable functionality in the terahertz band. Funct. Compos. Struct..

[22] Kronberger R., Wienstroer V. 3D-printed FSS using printing filaments with enclosed metal particles. Proceedings of the 2017 Progress in Electromagnetics Research Symposium—Fall (PIERS—FALL).

[23] Sanz-Izquierdo B., Parker E.A. (2014). 3-D Printing of Elements in Frequency Selective Arrays. IEEE Trans. Antennas Propag..

[24] Duan Y., Liang Q., Yang Z., Li Z., Yin H., Cao Y., Li D. (2021). A wide-angle broadband electromagnetic absorbing metastructure using 3D printing technology. Mater. Des..

[25] Lee S.-E., Lee W.-J., Oh K.-S., Kim C.-G. (2016). Broadband all fiber-reinforced composite radar absorbing structure integrated by inductive frequency selective carbon fiber fabric and carbon-nanotube-loaded glass fabrics. Carbon.

[26] Lee S.-E., Park K.-Y., Oh K.-S., Kim C.-G. (2009). The use of carbon/dielectric fiber woven fabrics as filters for electromagnetic radiation. Carbon.

[27] Whittow W., Li Y., Torah R., Yang K., Beeby S., Tudor J. (2014). Printed frequency selective surfaces on textiles. Electron. Lett..

[28] Mei H., Zhao X., Zhou S., Han D., Xiao S., Cheng L. (2019). 3D-printed oblique honeycomb Al_2_O_3_/SiCw structure for electromagnetic wave absorption. Chem. Eng. J..

[29] Yin L., Doyhamboure--Fouquet J., Tian X., Li D. (2018). Design and characterization of radar absorbing structure based on gradient-refractive-index metamaterials. Compos. Part B Eng..

[30] Tirado-Garcia I., Garcia-Gonzalez D., Garzon-Hernandez S., Rusinek A., Robles G., Martinez-Tarifa J., Arias A. (2021). Conductive 3D printed PLA composites: On the interplay of mechanical, electrical and thermal behaviours. Compos. Struct..

[31] Gorrasi G., Sorrentino A. (2013). Photo-oxidative stabilization of carbon nanotubes on polylactic acid. Polym. Degrad. Stab..

[32] Galos J., Hu Y., Ravindran A.R., Ladani R.B., Mouritz A.P. (2021). Electrical properties of 3D printed continuous carbon fibre composites made using the FDM process. Compos. Part A Appl. Sci. Manuf..

[33] Guo H., Zhao H., Niu H., Ren Y., Fang H., Fang X., Lv R., Maqbool M., Bai S. (2021). Highly Thermally Conductive 3D Printed Graphene Filled Polymer Composites for Scalable Thermal Management Applications. ACS Nano.

[34] Khodabakhshi S., Fulvio P.F., Andreoli E. (2020). Carbon black reborn: Structure and chemistry for renewable energy harnessing. Carbon.

[35] Bauhofer W., Kovacs J.Z. (2009). A review and analysis of electrical percolation in carbon nanotube polymer composites. Compos. Sci. Technol..

[36] Min Y.K., Eom T., Kim H., Kang D., Lee S.-E. (2023). Independent Heating Performances in the Sub-Zero Environment of MWCNT/PDMS Composite with Low Electron-Tunneling Energy. Polymers.

[37] Cao M.-S., Song W.-L., Hou Z.-L., Wen B., Yuan J. (2010). The effects of temperature and frequency on the dielectric properties, electromagnetic interference shielding and microwave-absorption of short carbon/fiber silica composites. Carbon.

[38] Kasgoz A., Korkmaz M., Durmus A. (2019). Compositional and structural design of thermoplastic polyurethane/carbon based single and multi-layer composite sheets for high-performance X-band microwave absorbing applications. Polymer.

[39] Lei L., Yao Z., Zhou J., Wei B., Fan H. (2020). 3D printing of carbon black/polypropylene composites with excellent microwave absorption performance. Compos. Sci. Technol..

[40] Gao Q., Ye X., Luo A., He E., Yang C., Yang P., Yan T., Ye Y., Wu H. (2023). 3D printing of carbon black/polylactic acid/polyurethane composites for efficient microwave absorption. J. Mater. Sci. Mater. Electron..

[41] Meng X.-M., Zhang X.-J., Lu C., Pan Y.-F., Wang G.-S. (2014). Enhanced absorbing properties of three-phase composites based on a thermoplastic-ceramic matrix (BaTiO_3_ + PVDF) and carbon black nanoparticles. J. Mater. Chem. A.

[42] Liu L., Duan Y., Ma L., Liu S., Yu Z. (2010). Microwave absorption properties of a wave-absorbing coating employing carbonyl-iron powder and carbon black. Appl. Surf. Sci..

[43] Liu X., Zhang Z., Wu Y. (2011). Absorption properties of carbon black/silicon carbide microwave absorbers. Compos. Part B Eng..

