# Supplementary material for: Fusedly Deposited Frequency-Selective Composites Fabricated by a Dual-Nozzle 3D Printing as Microwave Filter
Source: Polymers (Basel). 2024 Mar 12;16(6):786. doi: 10.3390/polym16060786 (PMC10974157; doi:10.3390/polym16060786)
Supplement: Supplementary file 1 [file polymers-16-00786-s001.zip › polymers-2599369-supplementary.pdf]

Supporting information

Fusedly Deposited Frequency-Selective Composites Fabricated by a Dual-Nozzle 3D Printing as Microwave Filter

Jae-Yeon Cho [1], Young-Chan Oh [1], Seung-Cheol Shin [1], Sun-Kon Lee [1], Hyoung-Seock Seo [2,3,*] and Sang-Eui Lee [1,*]

[1] Department of Mechanical Engineering, Inha University, 100 Inha-ro, Michuhol-gu, Incheon 22212, Republic of Korea
[2] School of Naval Architecture and Ocean Engineering, University of Ulsan, Ulsan 44610, Republic of Korea
[3] Department of Autonomous Vehicle System Engineering, Chungnam National University, 220 Gung-dong, Youseong-gu, Daejeon 305-764, Republic of Korea
* Correspondence: seohs@cnu.ac.kr (H.-S.S.); selee@inha.ac.kr (S.-E.L.); Tel.: +82-42-821-7223 (H.-S.S.); +82-32-860-7377 (S.-E.L.)

In order to fabricate FD-FSCs transmitting and absorbing microwaves in the X-band (8.2~12.4 GHz) range, the resonance frequency was calculated according to the design parameters of a metallic FSS. The approximate admittance predicting the resonant frequency of a metallic FSS [19] is as follows.

$$Y_{ind} \approx (-\mathrm{j})(\beta - \beta^{-1})\frac{\left[\left(\frac{A}{C}\right)+\frac{1}{2}\left(\frac{A}{\lambda}\right)^2\right]}{\ln\csc\left(\frac{\pi\delta}{2A}\right)} \quad \beta = \left(1-0.41\frac{\delta}{A}\right)\Big/\left(A/\lambda\right) \tag{S1}$$

Design parameters *A* and *B* are the side lengths of periodic patterns and apertures, and λ is the wavelength. δ is defined as (*A*-*C*)/2. At the resonance frequency, $Y_{ind} = 0$ and in the case of a metallic FSS, microwaves are completely transmitted. Y_{ind} depends only on two parameters A/λ and *C*/*A*. When $\beta - \beta^{-1}$ is zero, Y_{ind} is zero.

β for $\beta - \beta^{-1} = 0$ is obtained as follows.

$$\beta = \left(1-0.41\frac{\delta}{A}\right)\Big/\left(A/\lambda\right) = 1 \tag{S2}$$

$$\left(1-0.41\frac{\delta}{A}\right) = \frac{A}{\lambda} \tag{S3}$$

The resonance frequency is determined by the design parameters *A* and *C* of the inductive FSS, an aperture type of FSS with the conductive areas connected together. The resonance frequency (f_{res}) can be calculated from Equation (S3). Table S1 provides the calculated f_{res} according to the unit cell geometry of the FSS.

Table S1. Design parameters and resonant frequency of the metallic FSS.

A (mm)	C (mm)	δ (mm)	λ (mm)	f (GHz)
10.0	7.0	1.5	10.7	28.2
10.0	8.0	1.0	10.4	28.8
10.0	9.0	0.5	10.2	29.4
18.0	12.6	2.7	19.2	15.6
18.0	14.4	1.8	18.8	16.0
18.0	16.2	0.9	18.4	16.3
20.0	14.0	3.0	21.3	14.1
20.0	16.0	2.0	20.9	14.4
20.0	18.0	1.0	20.4	14.7
24.0	16.8	3.6	25.6	11.7
24.0	19.2	2.4	25.0	12.0
24.0	21.6	1.2	24.5	12.2
30.0	21.0	4.5	32.0	9.4
30.0	24.0	3.0	31.3	9.6
30.0	27.0	1.5	30.6	9.8

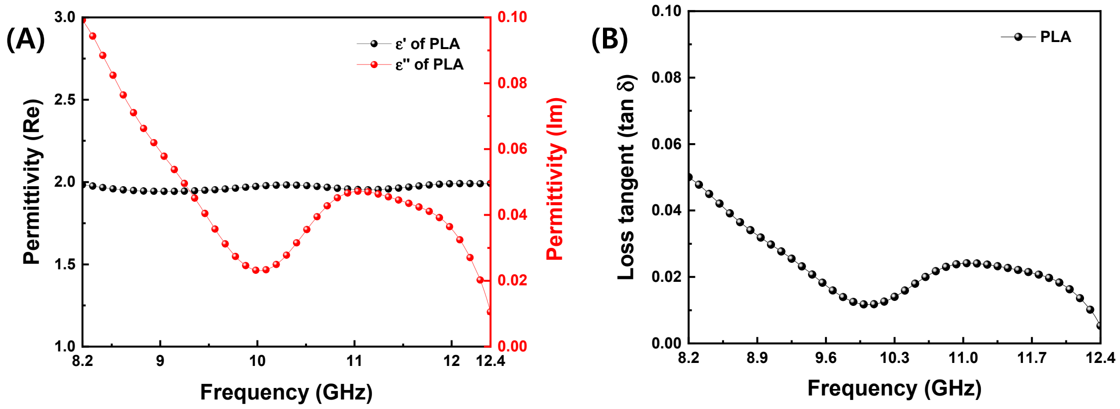

Figure S1. Measured permittivity of PLA. (A) Real and imaginary parts of permittivity, and (B) dielectric loss tangent.
